# Characterization of the vaginal microbiome of postmenopausal patients receiving chemoradiation for locally advanced cervical cancer

**DOI:** 10.1172/jci.insight.176839

**Published:** 2025-02-04

**Authors:** Brett A. Tortelli, Jessika Contreras, Stephanie Markovina, Li Ding, Kristine M. Wylie, Julie K. Schwarz

**Affiliations:** 1 Department of Radiation Oncology, Washington University School of Medicine, St. Louis, Missouri, USA.; 2 Transitional Year Program, Mercy Hospital St. Louis, St. Louis, Missouri, USA.; 3 Alvin J. Siteman Cancer Center,; 4 Department of Medicine,; 5 McDonnell Genome Institute,; 6 Department of Genetics,; 7 Department of Pediatrics, and; 8 Department of Cell Biology and Physiology, Washington University School of Medicine, St. Louis, Missouri, USA.

**Keywords:** Microbiology, Oncology, Cancer, Cervical cancer

## Abstract

The standard-of-care treatment of locally advanced cervical cancer includes pelvic radiation therapy with concurrent cisplatin-based chemotherapy and is associated with a 30%–50% failure rate. New prognostic and therapeutic targets are needed to improve clinical outcomes. The vaginal microbiome has been linked to the pathogenesis of cervical cancer, but little is known about the vaginal microbiome in locally advanced cervical cancer as it relates to chemoradiation. In this pilot study, we utilized 16S rRNA gene community profiling to characterize the vaginal microbiomes of 26 postmenopausal women with locally advanced cervical cancer receiving chemoradiation. Our analysis revealed diverse anaerobe-dominated communities whose taxonomic composition, diversity, or bacterial abundance did not change with treatment. We hypothesized that characteristics of the microbiome might correlate with treatment response. Pretreatment microbial diversity and bacterial abundance were not associated with disease recurrence. We observed a greater relative abundance of *Fusobacterium* in patients who later had cancer recurrence, suggesting that *Fusobacterium* could play a role in modifying treatment response. Taken together, this hypothesis-generating pilot study provides insight into the composition and dynamics of the vaginal microbiome, offering proof of concept for the future study of the microbiome and its relationship with treatment outcomes in locally advanced cervical cancer.

## Abstract 2

We characterize the vaginal microbiome of postmenopausal women with locally advanced cervical cancer undergoing chemoradiation.

## Introduction

Cervical cancer remains one of the leading causes of cancer and cancer death worldwide, with an average age at diagnosis of 53 years (1). Though the adoption of widespread screening has led to a decrease in cervical cancer rates in the United States, an estimated 4,000 people die from the disease annually (2). The treatment of cervical cancer is dependent on stage. Early-stage disease can be treated surgically or with radiation alone. The standard-of-care treatment of locally advanced cervical cancer includes pelvic radiation therapy with concurrent cisplatin-based chemotherapy and is associated with a 30%–50% failure rate (3). Recurrent cervical cancer is incurable and associated with high mortality (4); thus, new prognostic markers and therapeutic targets are needed to improve clinical outcomes.

The vaginal environment is a dynamic ecosystem, hosting various microbial species from diverse taxa including bacteria, fungi, and viruses (5–7). The healthy vaginal microbiome is often characterized by *Lactobacillus* dominance, which is believed to provide host protection against pathogens in part through the acidification of the vaginal microenvironment. However, while the vaginal microbiome of reproductive-aged women is frequently dominated by *Lactobacillus* species, the microbiomes of postmenopausal women have been shown to exhibit a lower abundance of lactobacilli with increased community diversity (8, 9).

There is a growing appreciation of the influence of the microbiome on the host immunity in the setting of cancer, which has driven interest in the potential of the microbiome as both a prognostic indicator and therapeutic target (10). While in some studies, perturbations of the vaginal microbiome have been linked to HPV infection and associated cervical diseases, the relationship of the vaginal microbiome to the development of cervical cancer and outcomes after standard-of-care treatments remains unclear (11). A few studies have characterized the composition and dynamics of the microbiome as it relates to chemoradiation in the setting of gynecologic malignancy (12–14). However, interpretation of these studies is complicated by the inclusion of patients who differ by menopausal status, gynecologic malignancy, and treatment approach. Furthermore, while the composition of the gut microbiome has been associated with survival in women treated for locally advanced cervical cancer (15), a relationship between the vaginal microbiome and treatment response has not been reported. In this pilot study, we aimed to specifically characterize the vaginal microbiome of postmenopausal women receiving standard-of-care chemoradiation to determine how the microbiome changed with therapy and whether any microbiome characteristics were associated with outcome.

## Results

### Study population.

Twenty-six women who are postmenopausal receiving intracavitary brachytherapy as part of the standard-of-care chemoradiation for locally advanced (Stage IB2 or greater) cervical cancer were included in our analysis. The average age of women in this study was 63 years, with a range of 52–77 years. The patient cohort was predominantly White (80.8%), with fewer Black (11.5%) and Asian (7.5%) patients. Most (84.6%) were diagnosed with squamous cell carcinoma, and most (84.6%) were positive for high-risk HPV. Initial-staging PET imaging showed 6 patients (23.1%) had positive pelvic lymph nodes and 9 (34.6%) had positive aortic lymph nodes. All but 1 patient was treated with cisplatin chemotherapy. Demographic and clinical characteristics of the patient cohort are presented in Table 1.

### Microbiome characterization.

**Figure 1:**
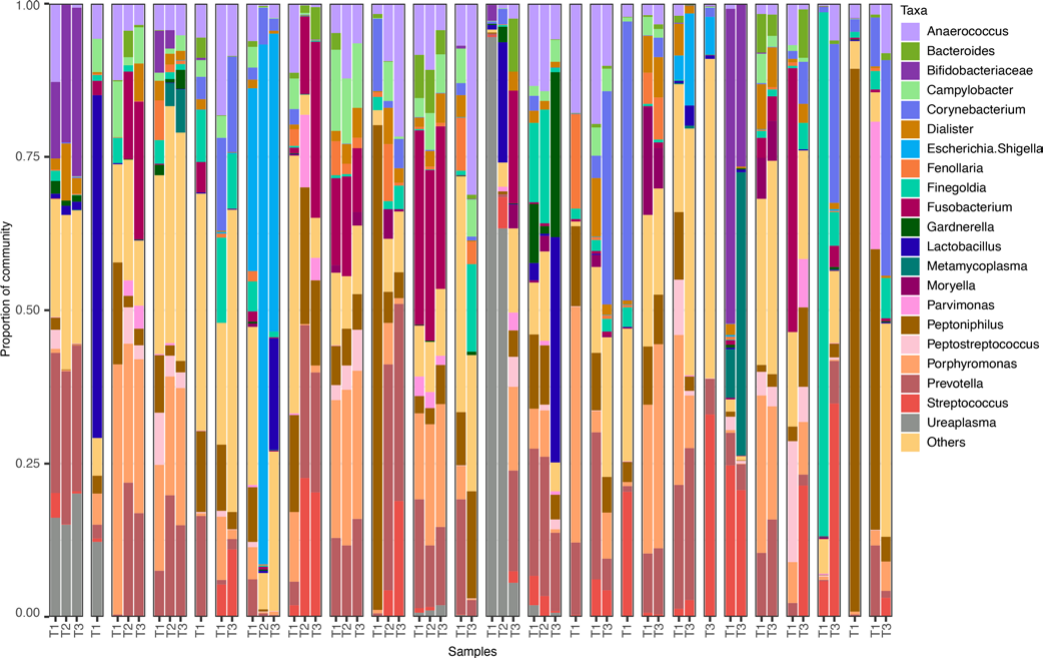
16S rRNA gene community profiles. Stacked bar graph showing the proportion of each taxa in a sample. Only the top 21 taxa identified within all samples are represented; all other taxa are grouped as “Others” for ease of visualization. Sampling time point (T1 = pretreatment, T2 = 1–2 weeks into treatment, and T3 = 3 weeks into treatment) is designated along the *x* axis and grouped by patient.

To characterize the composition of the microbiome, we performed 16S rRNA gene sequencing on 56 vaginal swab specimens collected from our cohort of patients. Of the 56 specimens, 25 were collected prior to chemoradiation (T1), and 31 were collected after chemoradiation was initiated; either 1–2 weeks after treatment start (T2) or 3 weeks after treatment start (T3). All but 5 T1 specimens and one T3 specimen had matched samples available from the same patient for comparison. We observed diverse communities with a median of 32 speciestaxa (interquartile range [IQR], 26–39) represented in each sample. Relative abundance data for each sample is presented in Supplemental Table 1 (supplemental material available online with this article; https://doi.org/10.1172/jci.insight.176839DS1). We generated stacked bar plots for each sample to illustrate the taxonomic composition of each community (Figure 1). *Lactobacillus* was uncommon, while anaerobes such as *Prevotella*, *Porphyromonas*, *Peptoniphilus*, *Anaerococcus*, and *Fusobacterium* were most abundant.

### Microbiome before and during chemoradiation.

**Figure 2:**
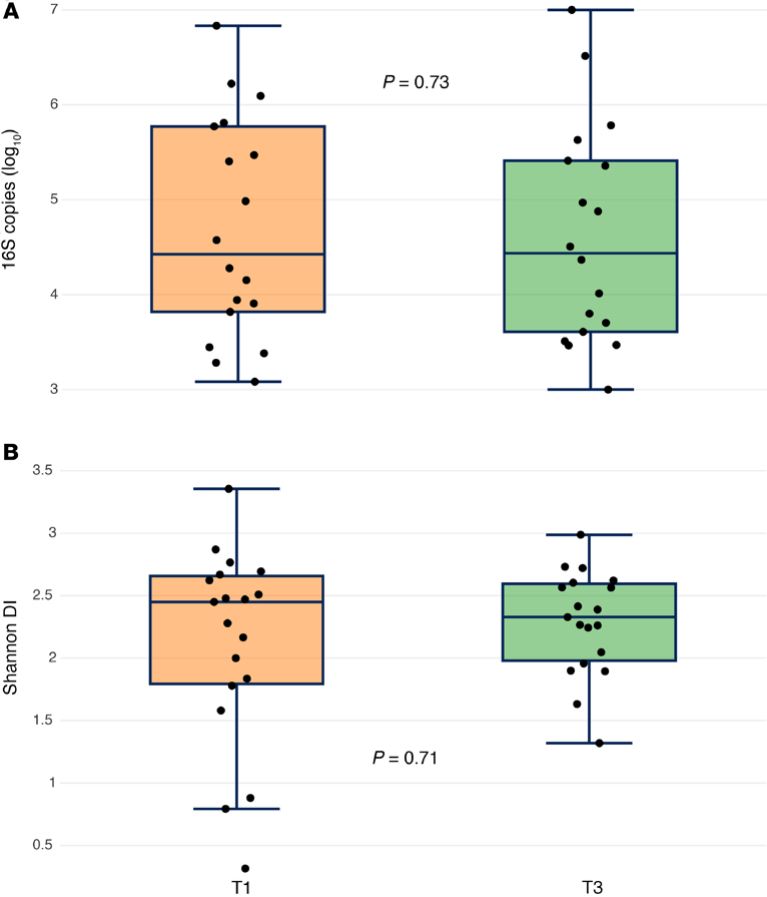
Bacterial abundance and α diversity by sampling time point. (**A** and **B**) A paired analysis of samples taken pretreatment (T1) and 3 weeks into treatment (T3) for bacterial abundance (**A**) and α diversity (**B**). *P* values were calculated with a Wilcoxon signed-rank test.

We compared the microbiome before and during chemoradiation therapy. To assess whether chemoradiation reduced the bacterial biomass we utilized a quantitative PCR (qPCR) assay to approximate the total bacterial abundance for each sample. In a paired analysis, we did not observe a significant difference in bacterial abundance before and during chemoradiation (*P* = 0.73) (Figure 2A). Among all samples, α diversity did not significantly differ by collection time point (*P* = 0.78). A paired analysis of T1 and T3 specimens also failed to show a difference in α diversity (*P* = 0.71) (Figure 2B).

**Figure 3:**
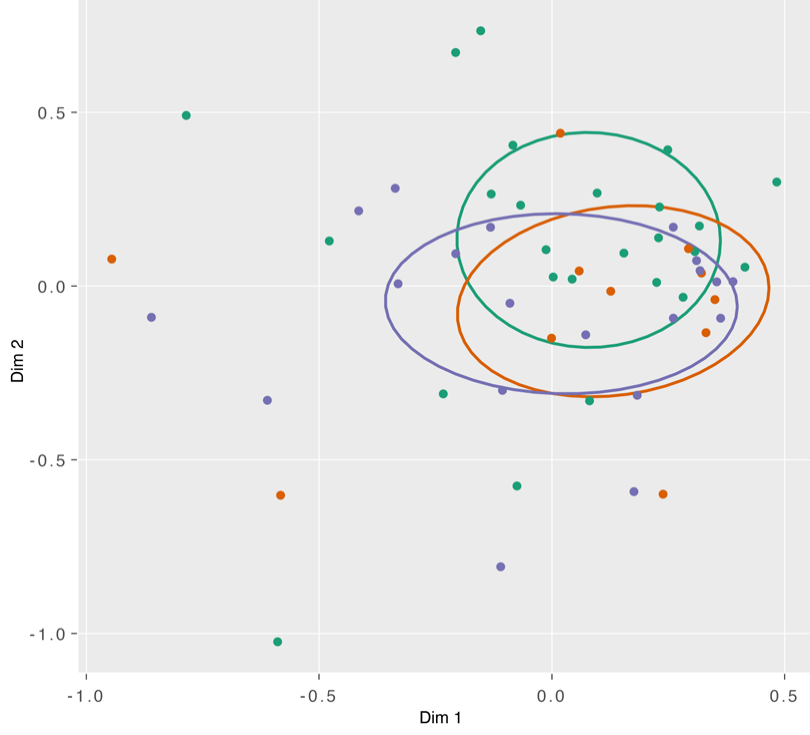
NMDS by time point. NMDS plot of taxonomic composition by sampling time point. Comparing samples taken prior to treatment (green), 1–2 weeks into treatment (orange), and 3 weeks into treatment (purple). Normal confidence ellipses represent 50% confidence level and centroid of data. Permutational multivariate ANOVA of distance matrices did not identify significant differences by sampling time point (*P* = 0.56).

Nonmetric dimensional scaling (NMDS) was used to plot and compare the similarity of the microbiome across individuals and sampling time points. Analysis of the NMDS data do not reveal any significant association between the taxonomic composition of the microbiome and sampling time point (*P* = 0.56). We observed that pretreatment and during-treatment samples collected from the same woman were more similar to one another than to samples collected from others at the same time point (Figure 3). To determine whether particular taxa were enriched or depleted by chemoradiation, we performed linear discriminant analysis (LDA) using patient microbiomes for which both T1 and T3 samples were available. However, we did not identify any notable enrichment or depletion of the relative abundance of taxa within this dataset. Analysis of compositions of microbiomes with bias correction (ANCOM-BC) is an alternative method for differential abundance analysis that corrects for sampling biases (16). We applied ANCOM-BC to the same dataset and confirmed the results obtained using LDA.

### Community characteristics by disease recurrence.

We sought to identify whether certain characteristics of the pretreatment microbiome were correlated with treatment response as determined by disease recurrence during the follow-up period. Pretreatment microbiome characterization and recurrence data were available for 24 of the patients. Eleven (46%) of the patients experienced a recurrence during the follow-up period. The median follow-up time for patients alive at time of last follow-up was 50 months (range, 20–61 months). We did not observe any association between demographic or clinical characteristics and disease recurrence. A summary of demographic and clinical characteristics by recurrence status can be found in Table 2.

**Figure 4:**
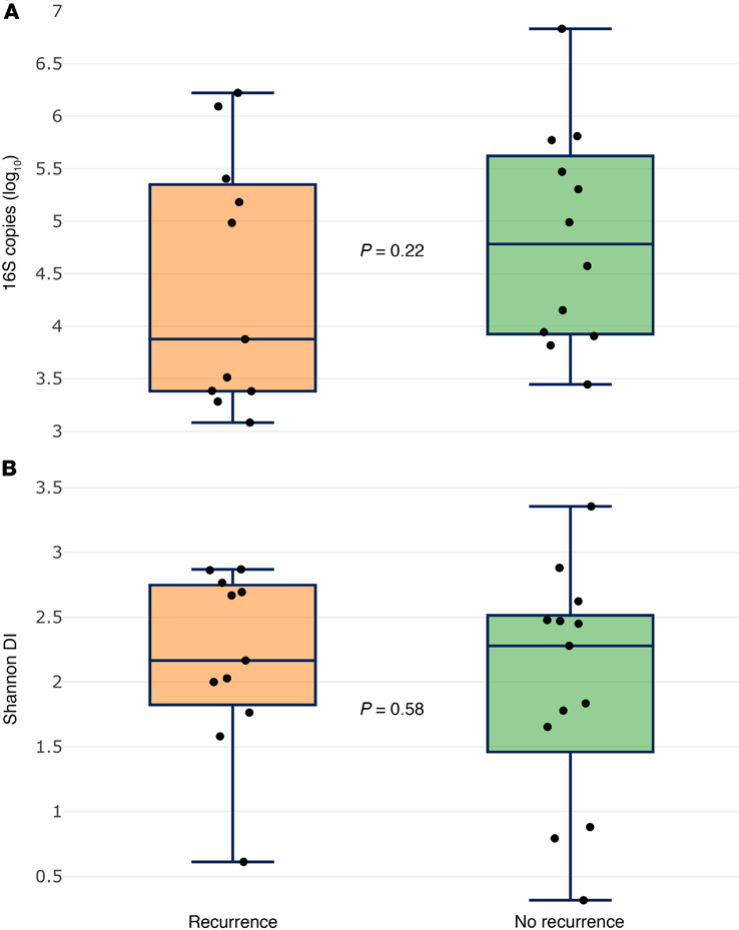
Bacterial abundance and α diversity by recurrence status. (**A** and **B**) An analysis of bacterial abundance (**A**) and α diversity (**B**) of pretreatment samples of patients who had disease recurrence and those that did not. *P* values were calculated with a Kruskal-Wallis rank sum test.

**Figure 5:**
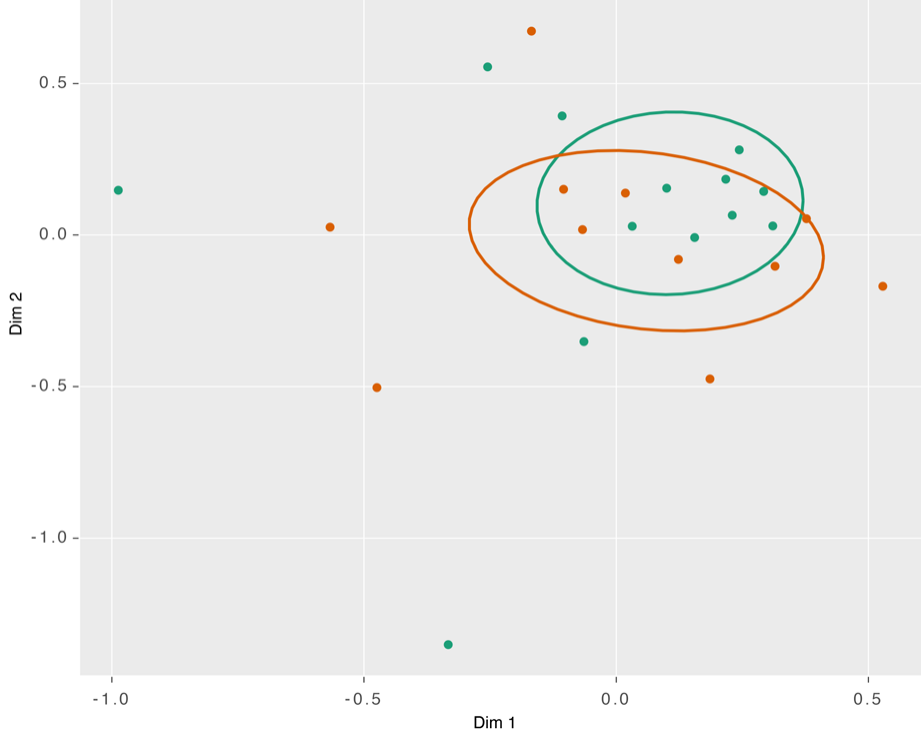
NMDS by recurrence status. NMDS plot of pretreatment taxonomic composition by recurrence status. Comparing samples from patients who had a recurrence (orange) and those who did not (green). Normal confidence ellipses represent 50% confidence level and centroid of data. Permutational multivariate ANOVA of distance matrices did not identify significant differences by sampling time point (*P* = 0.47).

**Figure 6:**
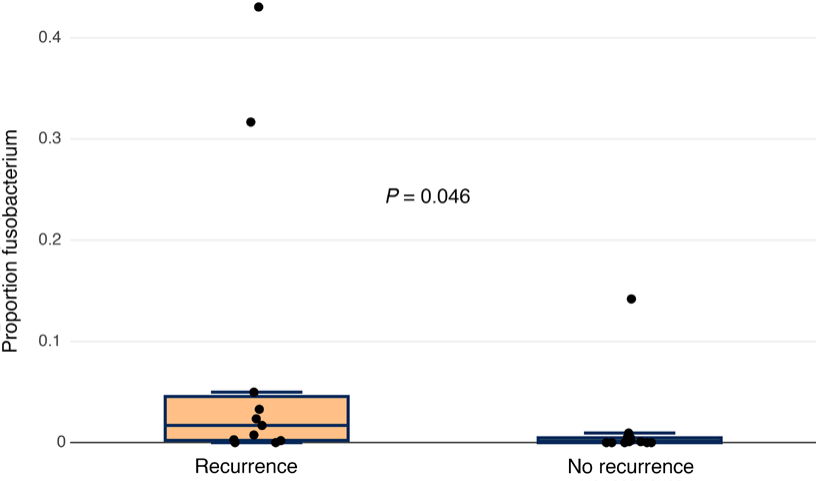
*Fusobacterium* abundance by recurrence status. The relative abundance of *Fusobacterium* in pretreatment samples of patients who had disease recurrence and those who did not. *P* values were calculated with a Kruskal-Wallis rank sum test.

We compared the pretreatment microbiomes of patients who developed disease recurrence (treatment nonresponders) to those who did not (treatment responders). There was no significant difference in bacterial abundance (*P* = 0.22) or community diversity (*P* = 0.58) between treatment responders and nonresponders (Figure 4). NMDS was used to plot and compare the microbiomes of treatment responders and nonresponders. Samples did not appear to cluster by recurrence status, and no significant difference was observed between treatment responders and nonresponders (*P* = 0.47) (Figure 5). In an effort to identify taxonomic biomarkers of treatment response within the microbiome, we correlated the relative abundance of the top 21 taxa with recurrence. Though it did not reach statistical significance, a greater relative abundance of *Fusobacterium* was observed among treatment nonresponders (median, 0.017; IQR, 0.002–0.041) than treatment responders (median, 0.001; IQR, 0.000–0.005) (unadjusted *P* = 0.046) (Figure 6). No other genera were identified to be enriched by treatment response (Supplemental Table 2). Linear discriminate analysis and ANCOM-BC corroborated these findings.

## Discussion

In this hypothesis-generating pilot study, we aimed to characterize the vaginal microbiome of postmenopausal women with locally advanced cervical cancer before and during standard-of-care chemoradiation therapy and identified whether microbial characteristics might be predictive of treatment response. We found that the microbial communities were diverse with few lactobacilli and an abundance of anaerobes. While we had hypothesized that treatment toxicities would alter the microbiota, enriching for chemo/radioresistant taxa, we did not identify characteristic changes in bacterial abundance, diversity, or taxonomic representation with treatment. Among our cohort, about one-third of the patients had disease recurrence within the follow-up period. While we did not identify any difference in bacterial abundance and community diversity based on disease recurrence, we did observe that the genus *Fusobacterium* was enriched in pretreatment samples in the patients who went on to have disease recurrence. This suggests that *Fusobacterium* may be predictive of disease recurrence and could play a role in modifying treatment response.

Several studies have demonstrated that the cervicovaginal microbiome is altered in the setting of gynecologic malignancy. They have shown that the microbiomes of patients with cervical cancer exhibit greater community diversity than the microbiomes of healthy controls (12, 14, 17). Furthermore, microbiomes of patients with cancer harbor less *Lactobacillus* than healthy controls, even in the postmenopausal setting where *Lactobacillus* is less common (12, 14). These communities instead have been shown to be enriched in anaerobic taxa associated with dysbiosis including *Prevotella*, *Porphyromonas*, *Peptoniphilus*, *Anaerococcus*, and *Fusobacterium* (12, 14). Our analysis revealed that the microbiomes of our cohort were similar to previously reported postmenopausal cancer cervicovaginal microbiomes with low prevalence of *Lactobacillus* and abundant anaerobes associated with dysbiosis. This adds confidence that the microbiomes observed within our cohort are representative of the communities found in the setting of cervical cancer more broadly.

Various physiologic and environmental factors have been shown to disrupt and alter the vaginal microbiome including menstrual cycle, menopause, pregnancy, hygiene, and sexual behavior (5, 18, 19). Since radiotherapy also exerts toxic effects on vaginal mucosa (20), we initially hypothesized that treatment would induce changes in the microbiome through mucosal and possibly microbial toxicities, leading to decreased bacterial diversity. But our analysis did not reveal any significant change in the richness or diversity of bacterial communities with radiotherapy. This finding is consistent with several small studies of patients with cervical cancer who did not identify significant changes in α diversity with radiotherapy (13, 14, 17). Though another study in a mixed cohort of patients with endometrial and cervical cancer reported an increase in diversity after radiotherapy (12).

We report that radiotherapy did not produce characteristic changes in community composition. When we looked at the abundant taxa in a community before radiotherapy and during radiotherapy, they appeared proportionally similar. There were a few communities that experienced significant substantial shifts in community composition, but the majority appeared relatively stable. Supporting this initial observation, our NMDS analysis did not identify any significant differences between samples based on collection time point. This finding is largely consistent with prior reports (12, 13, 17). Of note, 1 study of 16 patients with cervical cancer reported changes in community structure that were roughly able to distinguish between pre- and postradiotherapy microbiomes. Though this finding could be driven by differences in hormonal status within their cohort as they included both patients who are pre- and perimenopausal (14).

In addition to community shifts, we also hypothesized that particular taxa may be enriched after radiotherapy. However, our analysis did not reveal significant enrichment or depletion of taxa with treatment. This is in contrast to another study of postmenopausal patients with cervix and endometrial cancer that reported an enrichment of *Prevotella*, *Lachnospiraceae*, *Alistipes*, *Enterobacteriaceae*, and *Pseudomonas* in postradiotherapy samples (12). With the exception of *Prevotella,* these genera were rare in our cohort. Another analysis also reported the common vaginal microbiota genera *Streptococcus*, *Prevotella*, *Fusobacterium*, *Porphyromonas*, and *Finegoldia* were differentially abundant among pre- and postradiotherapy samples (14). While these genera were fairly well represented within our dataset, our analysis did not replicate this finding. Differences in patient demographics or methodology may explain these discrepancies.

Persistent HPV infection is associated with local immune suppression, which may influence the composition of the vaginal microbiome (21). Our results reported here are limited to the composition of the vaginal microbiome at the time of cancer diagnosis (which may occur decades after HPV infection) and during chemoradiation. At these time points, the effects of chronic HPV infection, advanced age, and menopausal status are difficult to separate, but it is clear that the combined effects are resulting in a dysbiotic vaginal microbiome that is different than the healthy postmenopausal microbiome (8, 9). Bacterial biomass is rarely quantitated in microbiome analysis but may be an important factor in host-microbe interactions in the setting of cancer. A reduction in bacterial quantity after radiotherapy was previously reported in a pilot analysis of pre- and postmenopausal patients with cervical cancer (13). However, our analysis did not reveal a significant change in bacterial burden with treatment, even in the setting of recommended douching.

There is increasing evidence that the microbiome modulates response to cancer therapies (10). A recent report indicated that gut microbiome diversity is an independent predictor of survival in patients with locally advanced cervical cancer treated with chemoradiation (15). However, when it comes to the relationship between the local cervicovaginal microbiome and outcomes, little is known. Here we report an enrichment of the genus *Fusobacterium* in pretreatment microbiomes of postmenopausal women who went on to have disease recurrence. While this pilot study could not demonstrate statistical significance, it raises the intriguing possibility that *Fusobacterium* may modify treatment response in this patient population.

*Fusobacterium* colonizes human mucosal sites and has been implicated in the pathogenesis of several malignancies including colorectal, oropharyngeal, and cervical cancers (22–25). Experimental models have shown that *Fusobacterium* is able to modify the tumor immune microenvironment and promote cancer cell proliferation, metastasis, and immune evasion (22, 23). *Fusobacterium* has even been associated with therapeutic response and survival. In locally advanced rectal cancer, *Fusobacterium* was found to be enriched in the baseline microbiomes of neoadjuvant chemoradiotherapy nonresponders (26). Abundant intratumoral *Fusobacterium* has been reported in patients with colorectal cancer recurrence after chemotherapy, and experimental evidence suggests that the bacterium is able to reduce chemotherapy-induced cancer cell apoptosis (27). In cervical cancer, intratumoral *Fusobacterium* quantity has been associated with more advanced stage cervical cancer and poor progression free survival (28). Similarly, intratumoral *Fusobacterium* in esophageal squamous cell carcinoma is associated with poor recurrence-free survival and poor chemotherapeutic response (29). Interestingly, *Fusobacterium* has not been associated with poor prognosis in all cancers. Two separate studies have found that intratumoral *Fusobacterium* quantity was associated with improved survival and lower rates of recurrence in oral squamous cell carcinoma patients (30, 31). Additional study will be needed to evaluate what role *Fusobacterium* might play in modifying therapeutic response in cervical cancer.

In conclusion, we showed that chemoradiation did not induce characteristic changes in the cervicovaginal microbiome of patients who are postmenopausal with locally advanced cervical cancer. We also identified *Fusobacterium* to be enriched in the pretreatment microbiomes of patients who are postmenopausal who went on to have disease recurrence after treatment. This study has several strengths. First, it leverages samples collected from a well-characterized institutional cohort receiving similar treatment for which outcome data were prospectively collected. We also limited our analysis to patients who are postmenopausal, reducing the potential confounding effect of induced menopause. A notable limitation of our study is the small sample size. Future studies in larger, well-powered cohorts will be needed to verify these findings and correlate cervicovaginal microbiome with intratumoral bacterial burdens and immune cell infiltration over the course of therapy.

## Methods

### Sex as a biological variable.

Due to the focus of this study on the vaginal microbiome, only female participants were examined.

### Study design.

Vaginal swab specimens were collected from patients receiving treatment for locally advanced cervical cancer (FIGO Stage IB2 or greater) at Washington University in St. Louis School of Medicine between 2017 and 2021. Treatment followed institutional guidelines and included external beam radiation with interdigitated intracavitary brachytherapy and concurrent cisplatin or carboplatin chemotherapy as previously described (32). Brachytherapy included weekly high dose rate (HDR) treatments for 6 weeks. One patient also received adjuvant pembrolizumab therapy as part of a clinical trial (33). During this time, women were instructed to use peroxide vaginal douches to reduce risk of infection (started after collection of the pretreatment specimen). Vaginal swab specimens were collected by providers at the initial consultation (T1) and at 2 time points during brachytherapy; either 1–2 weeks (T2) or 3 weeks into treatment (T3). A double dry rayon swab (Starplex Scientific) was inserted into the bilateral vaginal fornices and used to sweep anteriorly and or posteriorly behind the cervix. Vaginal swab specimens were stored at –80°C prior to processing as described below. Twenty-nine patients who are postmenopausal were identified for inclusion in this study.

### Clinical data.

Demographic and clinical data were collected from patients, and follow-up occurred at 6 weeks, 3 months, 6 months, 12 months, 2 years, and 3 years after the completion of chemoradiation. Clinical data were last updated in March of 2023.

### DNA extraction and 16S rRNA gene sequencing.

Genomic DNA was extracted from frozen vaginal swabs using QIAamp BiOstic Bacteremia DNA Kit (Qiagen). Sterile swab extractions were run in parallel as negative controls. 16S amplicon libraries were prepared with the *Quick*-16S DNA NGS Library Prep Kit (Zymo Research) using the 515F and 806R primer pair to amplify the V4 variable region (34). Indexed samples were then pooled and sequenced on the Illumina MiSeq platform. Paired-end 2 × 250 sequence reads were demultiplexed, quality filtered, and assigned to operational taxonomic units (OTUs) with taxonomic classification using an established pipeline based on the MOTHUR SOP (35). In brief, UCHIME was used to screen chimeric sequences (36), and taxonomic classification of the sequences was established using the naive Bayesian classifier (37) with the Ribosomal Database Project Training Set, version 18 (38). Classifications with confidence thresholds < 0.8 were reassigned to the next highest taxonomic level with a confidence > 0.8. The median read count for vaginal specimens was 1,496 reads, with an IQR of 1,296–1,837 reads. All samples with fewer than 1,000 assigned reads were removed from further analysis. This resulted in the removal of 9 of 65 samples. Rarefaction was utilized to normalize read counts across samples to 1,064 reads.

### Bacterial abundance quantification.

Genomic DNA was diluted 10-fold in sterile water and used to quantify the abundance of bacteria by qPCR. We were unable to quantify 1 sample included in our microbiome sequencing analysis due to insufficient biomaterial. Primers (515F and 806R) were used to quantify 16S rRNA gene V4 copy number (34). The 16S rRNA gene of *E*. *coli* was synthesized and used to create a standard from 1 × 10^3^ to 1 × 10^7^ copies per microliter. Samples quantified below or above the standard curve were assigned either the minimum or maximum standard value. Samples were run in duplicate using 2 μL of diluted genomic DNA for each qPCR. Each reaction was run with TB Green Advantage qPCR Premix (Takara Bio) on a 7500 Applied Biosystems Real-Time System with the following cycle conditions: 95°C for 5 minutes followed by 40 cycles of 95°C for 5 seconds, 55°C for 20 seconds, and 72°C 90 seconds.

### Data analysis and plotting.

All data analysis and plotting were conducted using RStudio Version 1.4.1717. Shannon diversity index (Shannon DI) was calculated with the package ‘vegan’ (39). We generated a Bray-Curtis dissimilarity matrix from the 16S rRNA gene taxonomic data and then performed nonmetric multidimensional scaling (NDMS) using the packages ‘vegan’ and ‘labdsv’ (39, 40). LDA was performed using the package ‘lefser’ with an effect size threshold of 1 (41). Differential abundance was assessed with the package ‘ANCOMBC’ (16). Data were plotted using the packages ‘plotly’ and ‘ggplot2’ (42, 43).

### Statistics.

All statistical analysis was conducted in Rstudio v1.4.1717. Wilcoxon signed-rank test, Kruskal-Wallis rank sum test, and Fisher’s exact test were used as appropriate. Permutational multivariate ANOVA of distance matrices was performed using the ‘adonis’ function in the R package ‘vegan’ (39). A *P* value less than 0.05 was considered significant. Bonferroni correction was used to account for multiple comparisons when evaluating the correlation between genus relative abundance and treatment response.

### Study approval.

Written informed consent was obtained for all participants. The study was approved by the Washington University School of Medicine IRB.

### Data availability.

Sequencing data are available in BioProject PRJNA1029035. Patient data are available in Supplemental Table 3. Supporting data values for all figures can be found in the Supporting Data Values file.

## Author contributions

The study was designed by JKS, KMW, LD, and BAT. SM, JC, and JKS collected clinical data and samples. BAT conducted experiments and prepared sequencing libraries. BAT and KMW performed all data analysis. The manuscript was prepared by BAT, JKS, and KMW with edits by coauthors.

## Supplementary Material

Supplemental tables 1-3

Supporting data values

## Figures and Tables

**Table 1:**
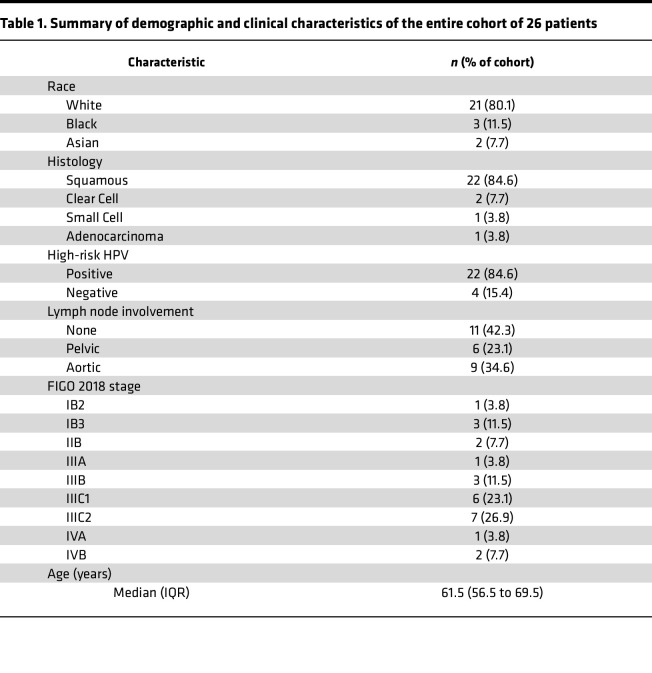
Summary of demographic and clinical characteristics of the entire cohort of 26 patients

**Table 2:**
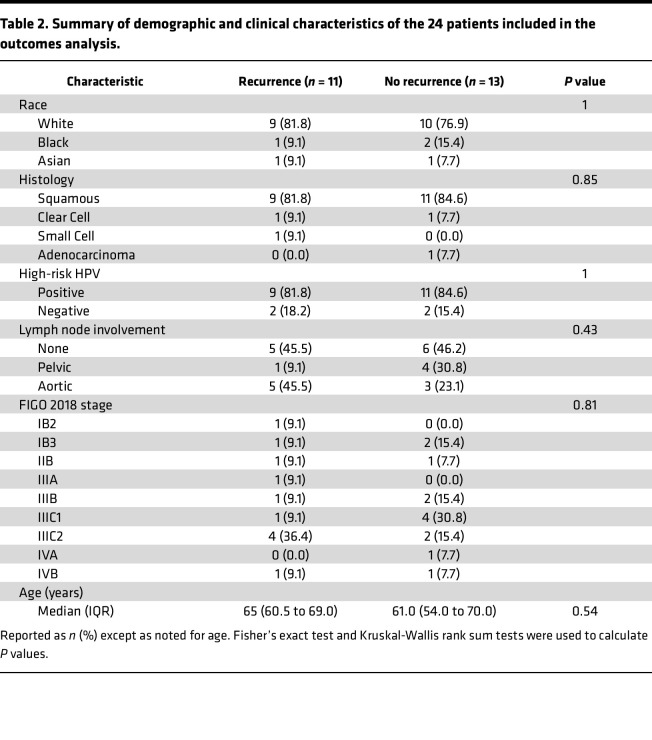
Summary of demographic and clinical characteristics of the 24 patients included in the outcomes analysis.

## Version 1 02/04/2025

In-Press Preview

## Version 2 03/24/2025

Electronic publication
